# Benefit of prokinetics during enteral nutrition: still searching for a piece of evidence

**DOI:** 10.1186/s13054-016-1502-3

**Published:** 2016-10-26

**Authors:** Alain Dive

**Affiliations:** Department of Intensive Care, Louvain School of Medicine, CHU UCL Namur, A. Dive, Avenue Therasse, 1, B-5530 Yvoir, Belgium

I read with interest the article by Lewis et al. [1] who presents a review and meta-analysis of randomized controlled trials (RCTs) examining the benefit and harms of prokinetics in patients receiving enteral nutrition. They used the Grading of recommendation, assessment, development, and evaluation (GRADE) method to assess the quality of evidence for various clinical outcomes including feeding tolerance, success of postpyloric placement tubes, pneumonia, mortality, vomiting, and diarrhoea.

It is important to note that this meta-analysis excluded studies comparing prokinetics to each other, and those assessing gastric emptying by pharmacokinetic (acetaminophen absorption) or isotopic methods. This may lead to important consequences.

Firstly, all the meta-analysed studies used gastric residual volume (GRV) as a marker of feeding (in)tolerance, with threshold values of 150 to 250 ml for the definition of intolerance. This may be considered obsolete since we know nowadays that GRV is a poor marker of gastric emptying, and the use of GRV for monitoring enteral nutrition was therefore recently challenged [2]. Taking this into consideration means that many patients included in this meta-analysis were falsely considered intolerant to enteral feeding. This does not contradict the main conclusions of the article, but instead it further weakens the already “moderate quality of evidence” that prokinetic agents are effective in improving feeding intolerance.

Another consequence of the study selection criteria is the relatively poor number of patients we are left with. Indeed, after separation of the articles assessing success of postpyloric tube placement or incidence of pneumonia, we are left with only five eligible RCTs and a total of 227 patients for evaluation of other major clinical outcomes including “feeding tolerance”, GRV, vomiting, and diarrhoea. Therefore, caution should be taken when interpreting the results. In particular, the increased rate of diarrhoea did not reach statistical significance, although the incidence of diarrhoea almost doubled in patients under prokinetic therapy in the two eligible studies. This is in accordance with a 40 % (single prokinetic) to 49 % (double prokinetic therapy) incidence of diarrhoea that was reported in patients fed enterally [3], suggesting the presence of digestive malabsorption aggravated by prokinetics in the critically ill. For intensive care unit (ICU) practitioners, “enteral feeding tolerance” is often assimilated to “adequate gastric emptying”. Although proper gastric emptying is a prerequisite for efficient enteral nutrition, the ultimate goal is to get the nutrients absorbed. In this regard, we may further question the place of prokinetics, as these may alter intestinal absorption through poorly adapted gut motility [4].

## Abbreviations

GRADE: Grading of recommendation, assessment, development, and evaluation
GRV: Gastric residual volume
ICU: Intensive care unit
RCT: Randomized, controlled trial
